# Skin Electrical Resistance as a Diagnostic and Therapeutic Biomarker of Breast Cancer Measuring Lymphatic Regions

**DOI:** 10.1109/access.2021.3123569

**Published:** 2021-10-27

**Authors:** NATASHA ANDREASEN, HENRY CRANDALL, OWEN BRIMHALL, BRITTNY MILLER, JOSE PEREZ-TAMAYO, ORJAN G. MARTINSEN, STEVEN K. KAUWE, BENJAMIN SANCHEZ

**Affiliations:** 1 IONIQ Sciences, Salt Lake City, UT 84103, USA; 2 Department of Electrical and Computer Engineering, University of Utah, Salt Lake City, UT 84112, USA; 3 Ogden Regional Medical Center, Department of Women’s Imaging, Ogden, UT 84405, USA; 4 Department of Physics, University of Oslo, 0371 Oslo, Norway; 5 Department of Clinical and Biomedical Engineering, Oslo University Hospital, 0372 Oslo, Norway; 6 Department of Materials Science and Engineering, University of Utah, Salt Lake City, UT 84112, USA

**Keywords:** Breast cancer, bioimpedance, skin resistance, machine learning

## Abstract

Skin changes associated with alterations in the interstitial matrix and lymph system might provide significant and measurable effects due to the presence of breast cancer. This study aimed to determine if skin electrical resistance changes could serve as a diagnostic and therapeutic biomarker associated with physiological changes in patients with malignant versus benign breast cancer lesions. Forty-eight women (24 with malignant cancer, 23 with benign lesions) were enrolled in this study. Repeated skin resistance measurements were performed within the same session and 1 week after the first measurement in the breast lymphatic region and non-breast lymphathic regions. Intraclass correlation coefficients were calculated to determine the technique’s intrasession and intersession reproducibility. Data were then normalized as a mean of comparing cross-sectional differences between malignant and benign lesions of the breast. Six months longitudinal data from six patients that received therapy were analyzed to detect the effect of therapy. Standard descriptive statistics were used to compare ratiometric differences between groups. Skin resistance data were used to train a machine learning random forest classification algorithm to diagnose breast cancer lesions. Significant differences between malignant and benign breast lesions were obtained (p<0.01), also pre- and post-treatment (p<0.05). The diagnostic algorithm demonstrated the capability to classify breast cancer with an area under the curve of 0.68, sensitivity of 66.3%, specificity of 78.5%, positive predictive value 70.7% and negative predictive value 75.1%. Measurement of skin resistance in patients with breast cancer may serve as a convenient screening tool for breast cancer and evaluation of therapy. Further work is warranted to improve our approach and further investigate the biophysical mechanisms leading to the observed changes.

## I. INTRODUCTION

Breast cancer is the most frequently diagnosed cancer in women and the leading cause of cancer deaths in women worldwide [1]. The goal of early breast cancer screenings is to increase the chance of survival and provide treatment early in the disease timeline. The current gold standard technique for breast cancer screening is mammography. Although mammography has had significant impact on screening for breast cancer, there are limitations in the accuracy of mammography and the appropriate age range for breast cancer screening still remains a matter of debate [2]. Annual mammograms are typically given to women above the age of 40–50 years old due to exposure of radiation and frequency of false positive results [3]. Because of this, many younger women are not screened for breast cancer until later in their life. Despite these limitations and issues with high costs, patient pain, and accessibility, mammography still remains the standard of care.

Breast cancer post-treatment monitoring also has its limitations [4]. The American Society of Clinical Oncology’s current guidelines with post-treatment breast cancer surveillance state that breast cancer survivors should continue to get annual screening mammograms with no routine laboratory tests or imaging [5]. The Cochrane review states that although more intensive post-treatment surveillance does not provide benefits of detecting recurrence or metastasis most breast cancer survivors prefer more frequent visits in order to be reassured about their health [6]. Due to the exposure of radiation, breast cancer survivors cannot obtain more frequent mammograms to monitor cancer recurrence [7]–[9]. With these limitations, an adjunctive technology to mammogram that provides noninvasive risk stratification may provide substantial benefit for patients unable to receive frequent mammograms or breast cancer survivors to monitor post-treatment progression.

Research suggests that cancer stimulates a cascade of physiological changes to the tissue matrix including interstitial fluids, lymph as well as cellular and anatomical structures [10]–[13]. These physiological changes may include triggering the system immune response, unbalanced ionic concentrations, biochemical expression, proteome changes, cellular differences, and altered tissue architectures. These changes within the interstitial and matrix and lymph system have been shown to be significant and measurable due to the presence of cancer in the body. The mechanism has been attributed to the high water and sodium content within cancerous tissues with movement of potassium, magnesium and calcium out of the cell [13]. Other possible contributors include altered membrane composition, nucleus-to-cytoplasm ratio and cellular composition and density.

Measuring electrical properties associated with these physiological changes that occur when cancer is present in the body has the potential to serve as an adjunctive technology that can provide early predictive diagnosis for patients with malignant breast lesions [14]. Bioimpedance is particularly well suited for the detection of physiological and structural changes in tissue since it is influenced by key parameters such as electrolyte concentration, pH, hydration state, and cell size and number [15]. Hence, bioimpedance can be used to distinguish between different tissue types or to detect pathological changes in tissue including the detection of skin [16], thyroid [17], oral [18], liver [12], cervix [19], [20], and prostate cancers [21].

In this study, we did not aim to measure the tumor tissue directly, but rather to measure the changes in skin resistance that occur from the body’s systemic immune response from cancer in the Sappey’s Plexus, an area of concentrated (high density) lymph capillaries below the nipple and areolar region [22], and non-breast lymphatic regions throughout the upper body. This study provides a description of our noninvasive approach, its successful application in patients with breast cancer at the clinic including inter-/intra-session reproducibility and tolerability data [23], and finally, an impedance machine learning algorithm to diagnose breast cancer and detect therapeutic effect [24], [25].

This paper consists of 6 sections. Section II discusses the materials used to conduct the study described. Section III describes the study methods including subject information, how the measurement procedure occurred, data analysis, and clinical methods of diagnosis. Section IV describes the results and assess the performance of the study. Sections V and VI discuss the interpretation of results and summarize the main conclusions, respectively.

## II. MATERIALS

### A. BIOIMPEDANCE MEASUREMENT DEVICE

**FIGURE 1.**
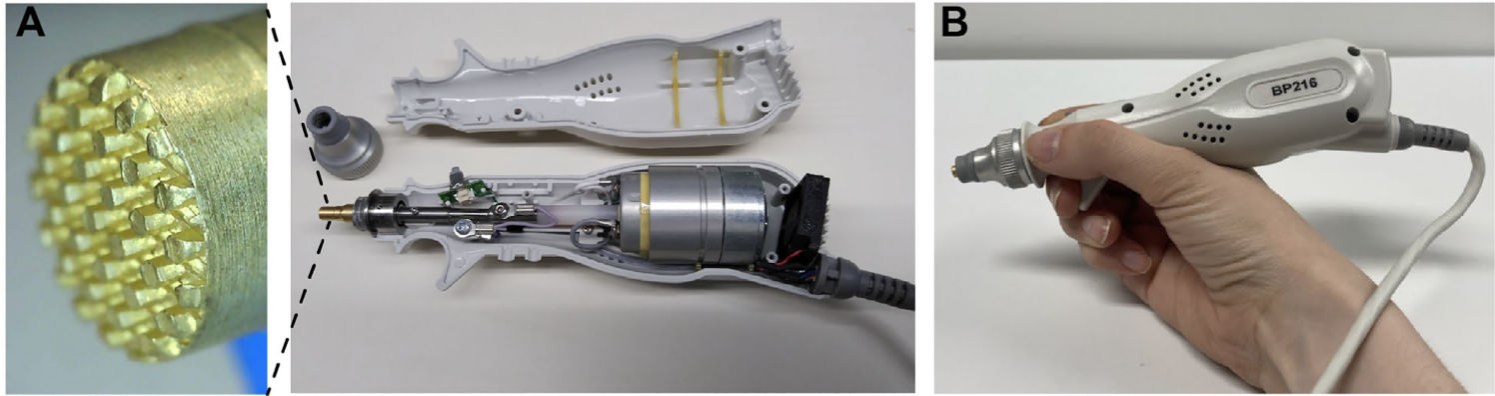
(A) Open view of the operator handheld signal probe showing the voice coil motor-controlled and electrode tip and closeup picture of the textured face of the brass signal electrode tip. (B) Use example of the handheld probe.

The device used to measure the bioimpedance for this study is a two-electrode DC system (IONIQ Sciences, Salt Lake City, UT). The system consists of an operator held probe housing through which a linear coil motor controls the position and force applied by a centrally located signal electrode with a disposable textured brass signal electrode (Figure 1), a reusable subject held reference electrode, and electronic board to generate current and measure the voltage signal, and a pulse-width modulation controller to apply the prescribed force profile to the hand held probe electrode. The textured electrode in the probe provides a fixed 2.35 ± 0.01 Volt DC signal and the current is limited to 25 *μ*A. The software guides the operator to moisten the skin under the handheld electrode and at the skin sites probed by the operator held probe.

The included animation video in the Supplementary Material is a demonstration of the probe measuring resistance values at an anatomical location. The displayed curve of the corresponding measurement produces a scaled conductance value from the resistance value for the ease of the operator. A high conductance value displayed correlates with a low resistance value and vice versa.

### B. ELECTRODES

The handheld probe has an overall length of 18.5 cm with a maximum diameter of 4 cm with a weight of 280 g. Inside the probe handle is a conductive shaft driven by a voice coil linear motor, and a cooling fan. The shaft is threaded which allows the operator to attach the textured disposable tip. During operation, the device applies a nominal force of 5.5 N to the textured tip onto the skin. The operator pushes the probe tip onto the skin with a force that must exceed this probe force. The electrode tip is surrounded by a small annular shroud. The operator pushes and holds the outer annular tip flush with the skin while the coaxially located electrode automatically extends and ramps up the force to the set level. The diameter of the textured tip is 4.5 mm with small hexagonal protrusions with a diameter of 0.54 mm. The subject handheld brass ground electrode is a cylinder with a diameter of 2.5 cm and length of 7.6 cm.

### C. DEVICE MEASUREMENT ACCURACY

The device used to measure resistance accuracy is a RS-200W Resistance Substituter range: 0–99,999,999.9 Ω in 0.1 Ω steps, accuracy ± 1% + 0.036 Ω, zero resistance: ≤0.5 Ω manufactured by IET Labs, Inc (Roslyn Heights, NY). Reference resistance measurements were performed from 1 to 100 kΩ in increments of 1 kΩ and then from 100 to 370 kΩ in increments of 10 kΩ.

## III. METHODS

### A. SUBJECT INFORMATION

**FIGURE 2.**
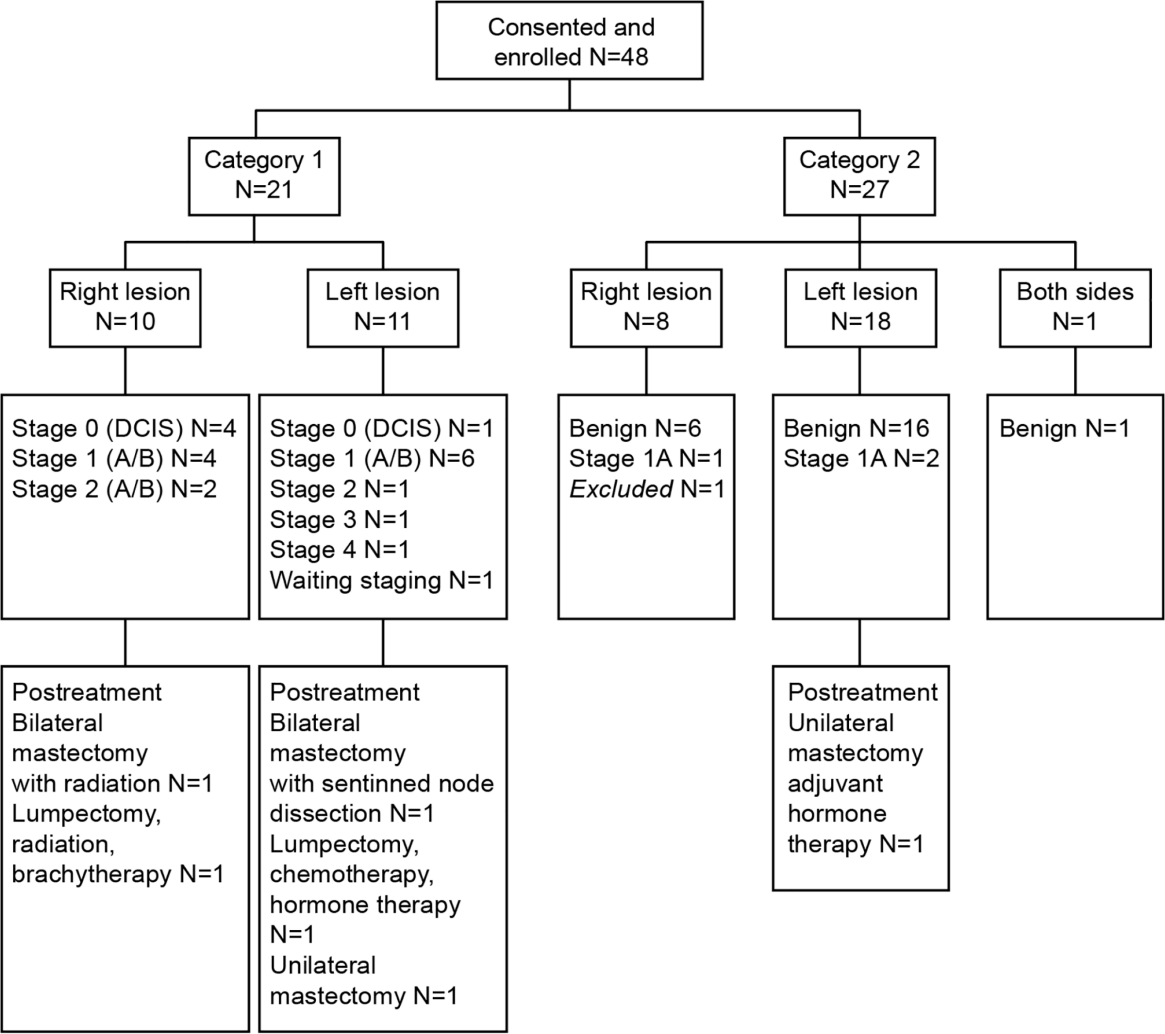
Study enrollment flow chart. Abbreviation: DCIS, ductal carcinoma in situ.

A total of 48 patients were studied through referral of a breast surgeon or oncologist (see study enrollment in Figure 2). The inclusion criteria for the patients included subjects to be females older than the age of 18. Among eligible patients, the following criteria disqualified the subject from the measurement protocol: Subject had an implanted electronic device in the chest, subject presented with an anomalous physical or anatomical condition that precludes the measurements, subjects had undergone unusually strenuous exercise within the last 24 hours, or the subject had significant systemic diseases such as uncontrolled diabetes, advanced heart failure, or a recent myocardial infarction, or other medical condition such as severe morbid obesity. A total of 1 patient was excluded from our analysis because the patient’s breast lesion does not have a confirmed pathological diagnosis. Each subject was enrolled in one of two categories:

- Category 1: Subjects with confirmed pathological diagnosis of cancer that have not yet received treatment for cancer (N = 21, mean age = 61.14 years, std = 11.39 years).
- Category 2: Subjects with a breast lesion that are indicated for a tissue biopsy, have not been pathologically confirmed for cancer and have not yet received treatment for cancer (N = 26, mean age = 53.23 years, std = 14.53 years).

### B. BIOIMPEDANCE MEASUREMENT

**FIGURE 3.**
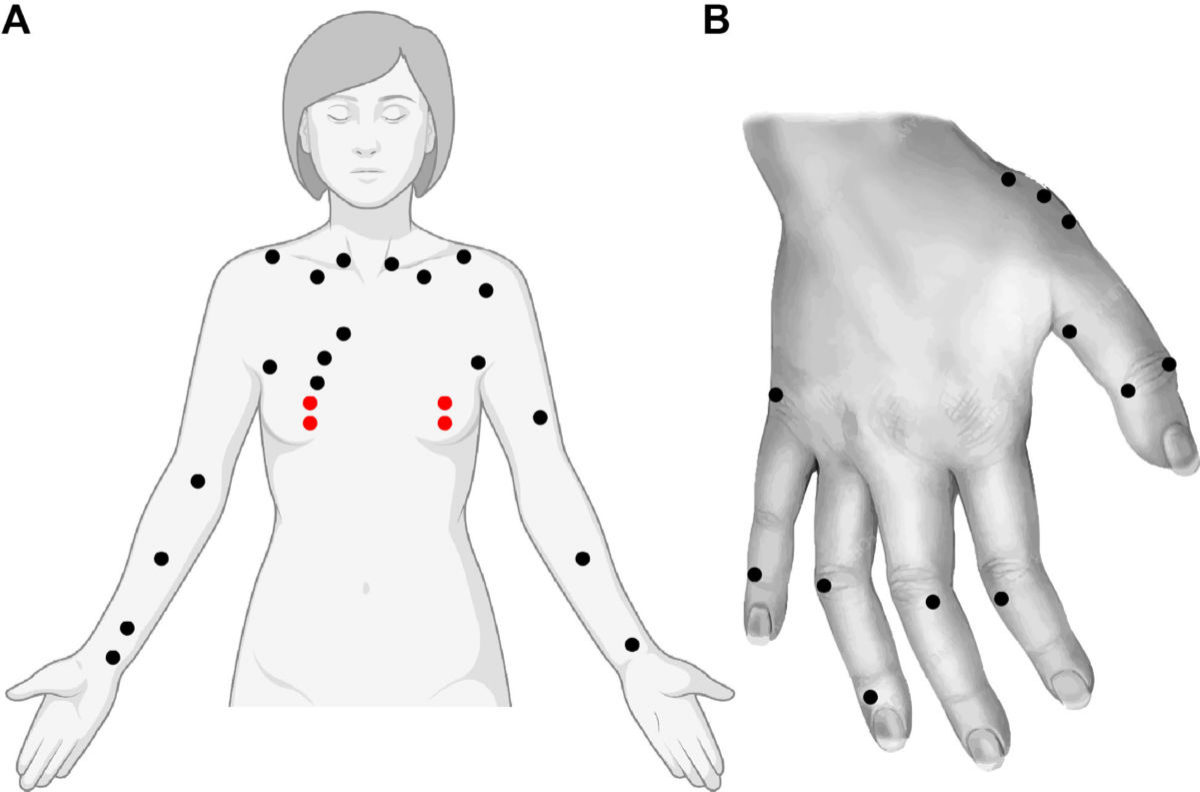
Schematic showing the bioimpedance measurement locations, non-breast lymphatic points (black) concentrated lymphatic Sappey’s Plexus (red). A total of 47 locations were measured in the measurement procedure including 4 concentrated lymphatic points and 43 less concentrated lymphatic control points located along the ribcage, collarbone, upper and lower arm, wrists, and fingers for both sides of the patient. Measurement points in the left hand not shown are reflected from the right hand.

The system measures the resistance between the location on the body that the operator places the probe tip, and the handheld ground electrode (see Figure 3 for locations measured in this study). The device begins recording as soon as the probe tip is touched to the skin. Simultaneously the voice coil motor algorithm is activated, and the probe tip force increases in a controlled ramp up to the control level of 5.5 N. The device monitors the signal resistance and holds the electrode tip in place for a controlled time based on the stability of the signal. At the end of the signal acquisition period, the probe tip motor is deactivated, and signal recording is terminated. Then the operator moves the tip to measure the next predefined anatomical location prompted by the software interface, moistens the skin as detailed in the protocol and takes the next measurement.

### C. EXPERIMENTAL PROTOCOL

This study was performed at Ogden Regional Medical Center (Ogden, UT) under an Institutional Review Board approved protocol (NCT04134520, October 22, 2019). The system was used as an investigational device. Written informed consent was obtained from each patient prior to measurements. Participants were compensated for the primary visit and any subsequent visit. Patients were measured with the bioimpedance system by the operator. In order to avoid confounding effects on the bioimpedance measurements, patients were instructed not to apply lotion or undergo unusually strenuous exercise for 24 hours prior to measurement procedure. All patients had received a mammogram confirming a lesion prior to measurement with the bioimpedance device.

Before the bioimpedance measurement procedure, the patient’s age, sex, height, weight, race/ethnicity, carcinogen exposure, medical history, and medications were recorded. The patient’s diagnosis was obtained through pathological diagnosis completed by staining biopsied tissue (hematoxylin and eosin and p63 stains) and applicable de-identified medical records were collected following Health Insurance Portability and Accountability Act guidelines.

During the bioimpedance measurement, the patient was lying in a supine position and reclined approximately at a 45 angle. The patient’s palms were prepped by hydration with water before gripping the handheld ground electrode. Each anatomical point location was also hydrated with water before the hand-held probe was applied. The probe was then placed by the operator sequentially at specific anatomical locations. A total of 47 locations were measured in the measurement procedure including 4 breast lymphatic points in the Sappey’s Plexus and 43 non-breast lymphatic points located throughout the upper body (see Figure 3). The less localized control points were located along the ribcage, collarbone, upper and lower arm, wrists, and fingers for both sides of the patient. The breast lymphatic points were located on the patient’s nipple directly superior and inferior to the nipple within in the areola on both breasts classified as Sappey’s Plexus.

Finally, a tolerability questionnaire was given which collected the patient’s subjective experience of discomfort. The questionnaire recorded whether the patient experienced discomfort (Yes or No), whether the patient would undergo the measurement again, and suggestions for improvement. If the patient indicated discomfort, comments on the type of discomfort, and a pain rating (scale 0–10) were recorded. Additional comments were also recorded. The overall measurement protocol session including the tolerability questionnaire took approximately 45 minutes per patient.

### D. DATA ANALYSIS

Resistance data measured from breast lymphatic points (N = 4) and non-breast lymphatic points located throughout the upper body (N = 43) were averaged and mean values normalized to generate individual data points for each patient.

### E. MAMMOGRAPHY

Routine digital two-dimensional bilateral craniocaudal (CC) and mediolateral oblique (MLO) images were obtained. R2 CAD software (Hologic, Inc., Marlborough, MA) was utilized to analyze the images. Separate bilateral three-dimensional tomosynthesis images in CC and MLO projections were obtained and reviewed. Included in the radiology report was the breast composition breast imaging-reporting and data system (BI-RADS) score and radiological findings, impression, and recommendations.

### F. PATHOLOGICAL DIAGNOSIS

For patients enrolled in Category 2, a pathological diagnosis was obtained through a clinical pathologist at the Ogden Regional Medical Center laboratory (Ogden, UT) which is certified under the Clinical Laboratory Improvement Act. A needle core biopsy was performed, and formalin fixed specimens were sent to pathology for staining. A hematoxylin and eosin stain was performed on the biopsy tissue as well as a p63 immunostain for myoepithelial cells. For patients enrolled in Category 1, these pathological diagnosis procedures were completed prior to the bioimpedance measurements.

### G. TREATMENT

A total of 6 patients were measured with the bioimpedance system both before and after receiving treatment for breast cancer. Post-treatment measurements were performed on average 6 months after treatment. For patients who returned post-treatment, treatment types and post-treatment de-identified medical records were collected. Their treatments are described below.

- Patient 1: Bilateral mastectomy with tissue expanders placed for future reconstruction surgery (reconstructive surgery not yet completed at time of post-treatment measurements). Right chest wall radiation receiving 50.4 Gray Gy) in 28 fractions. No chemotherapy or hormone therapy was required.
- Patient 2: Bilateral mastectomy with reconstruction and left sentinnel node dissection.
- Patient 3: Left side mastectomy. Adjuvant hormone therapy was started and will continue for 5 years. Patient received tamoxifen 20 mg daily. No reconstructive surgery done or planned. No radiation therapy or chemotherapy was required.
- Patient 4: Left breast lumpectomy. Brachytherapy was completed receiving 340 cGy in 10 fractions. Chemotherapy was completed. Patient started taking hormone therapy receiving anastrozole 1 mg daily.
- Patient 5: Left mastectomy with bilateral implant removal and right sentinel lymph node dissection. No radiation, adjuvant hormone therapy, or chemotherapy was required.
- Patient 6: Right breast lumpectomy. Adjuvant radiation Treatment/Brachytherapy was completed receiving 32 Gy in 8 fractions. No chemotherapy or hormone therapy was required.

### H. INTRASESSION AND INTERSESSION REPRODUCIBILITY ANALYSIS

Individuals underwent repeated testing to determine intrasession (N = 48) and intersession (N = 26) reproducibility by the intraclass correlation coefficient (ICC). Intersession measurements were determined on patients that returned for second measurement on average 1 week after to first measurement procedure. All reproducibility measurements were repeated twice by the first author (N.A.) and trained nurse examiner (B.M.).

### I. DATA ANALYSIS

Standard descriptive statistics were used to compare ratiometric differences between benign and malignant cancer groups (Prism, GraphPad, La Jolla, CA). Normalized averaged resistance values between non-breast and breast lymphatic hotspots are presented as individual data points, mean and standard error of the mean. A ratio closer to 1 would indicate that the breast lymphatic measurement was similar in value to the other non-breast lymphatic values in resistance. A value below 1 would indicate that the breast lymphatic values were higher than the non-breast lymphatic values in resistance. For ratiometric comparisons of changes among groups, t-test was used with Welch’s post-hoc test correction (two-tailed, significance 0.05).

### J. MACHINE LEARNING CLASSIFICATION ALGORITHM

We composed patients vector representations from three components: skin resistance represents the maximum resistance value and the change of resistance value of patient measurements for the list of points measured, patient age, and patient information including weight, height, body mass index, and smoking status. We quantified the model performance using these parameters by training a random forest model. The model was trained and evaluated using a leave-one-out cross-validation approach to ensure that our metrics represent out of sample performance. In order to accommodate for the stochastic nature of random forest model, we trained and evaluated 100 random forest models. The potential clinical utility was evaluated by constructing receiver-operating characteristic curves and extracting merit figures including area under the curve (AUC), sensitivity, specificity, positive predictive value (PPV) and negative predictive value (NPV).

## IV. RESULTS

### A. PATHOLOGICAL ASSESSMENT

**FIGURE 4.**
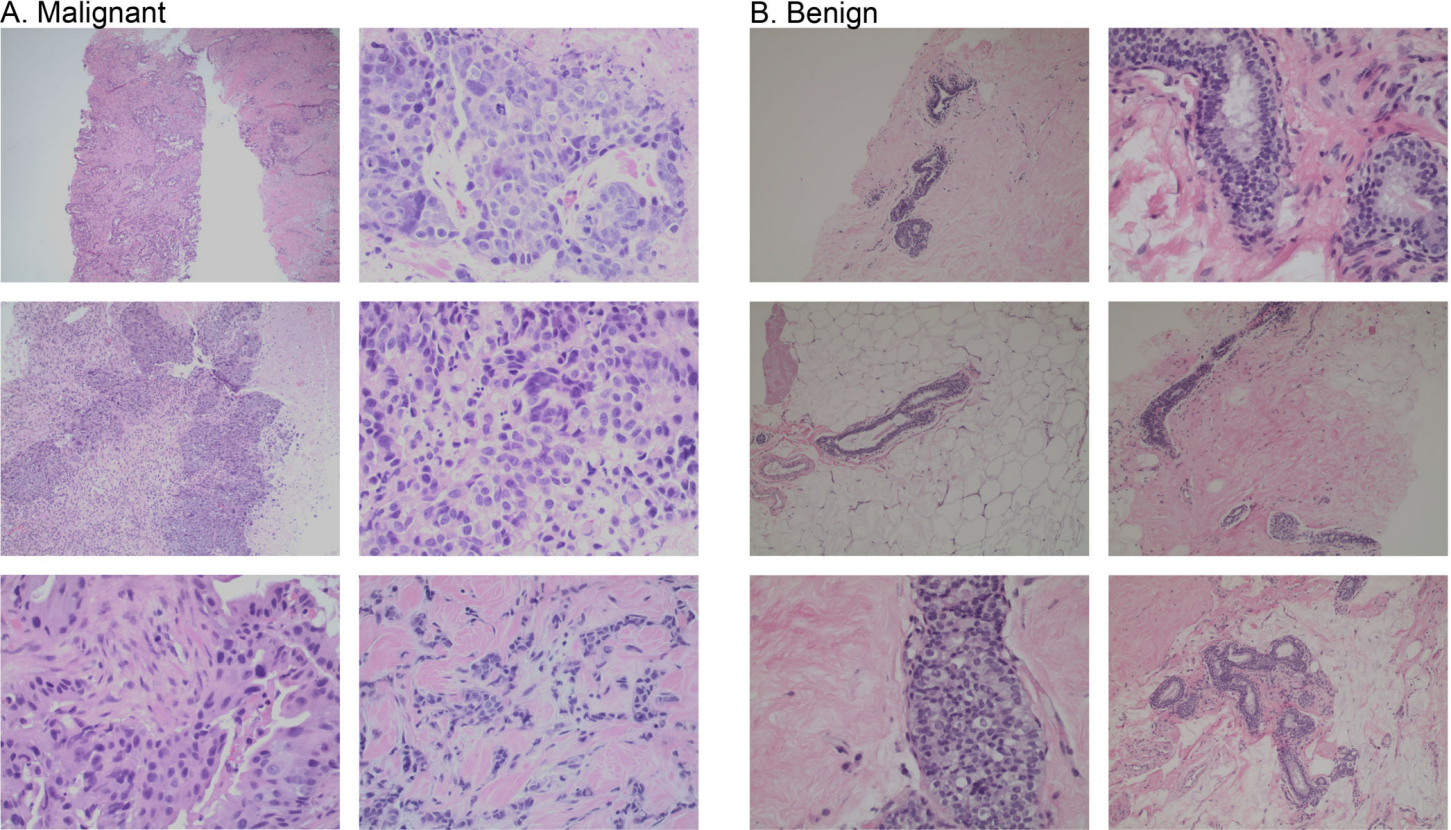
Representative histological findings from six patients with malignant (A) and benign (B) breast lesions.

Table 1 summarizes cancer patient characteristics and Figure 4 shows representative histology images of subjects with malignant and benign breast lesions. Malignant tissues in Figure 4 A show distortion in typical breast architecture, such as jagged borders and higher density of nuclei. Invasive breast carcinoma is distinguished by infiltrating nests of carcinoma cells and stromal infiltration by cords of small carcinoma cells showing a single file pattern as seen in the histology images. This contrasts with the benign pathology images that have a lower density of nuclei and smoother borders as seen in the Figure 4 B.

### B. DEVICE MEASUREMENT ACCURACY

**FIGURE 5.**
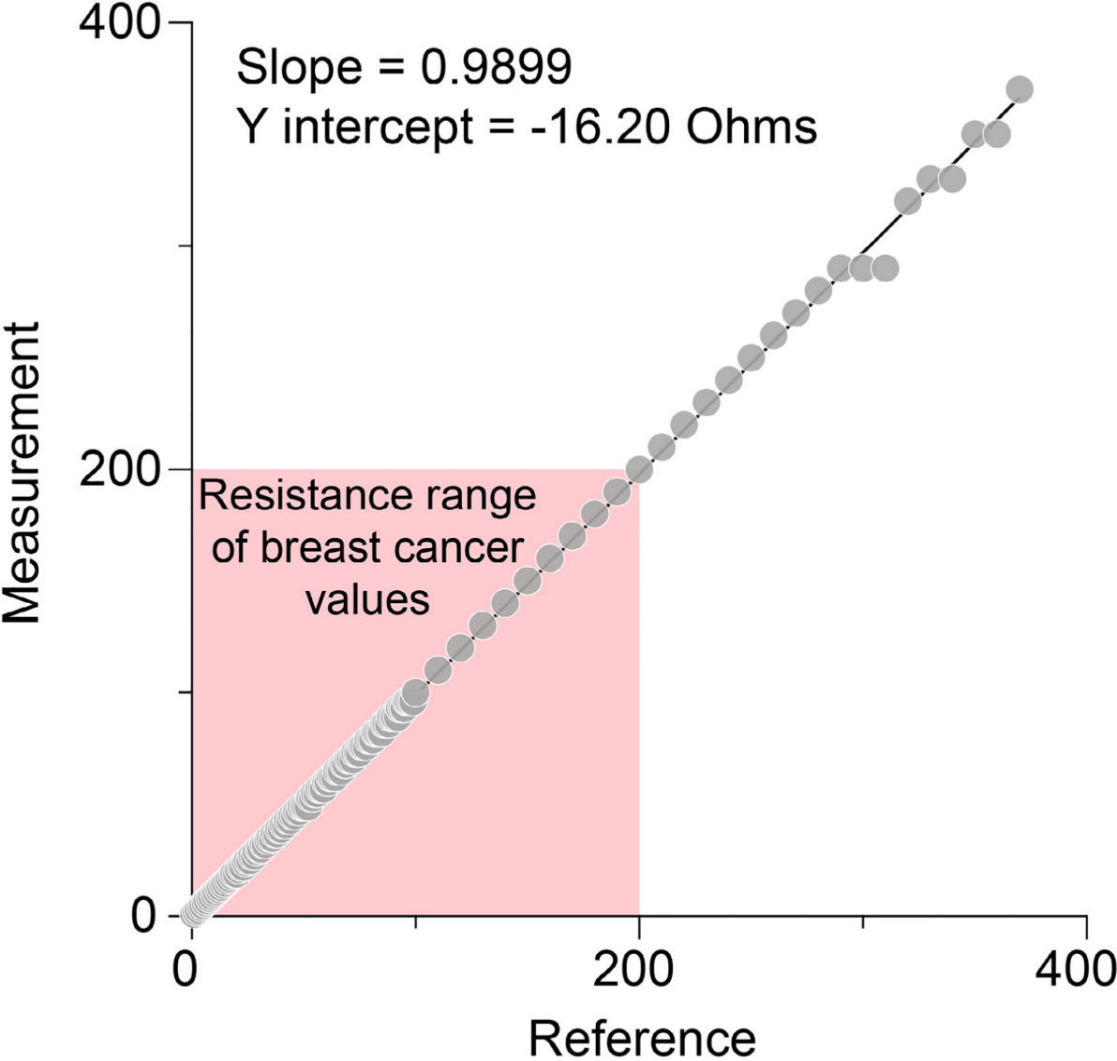
Measured versus reference resistance data (in kΩ) demonstrates high linearity over the dynamic range measured in patients with breast cancer.

The measured versus actual resistances of the reference device are shown with circles in Figure 5. A line was fitted for the resistance. The resistance measurements made by the device were linear (slope 0.9899 and y-intercept -16.20 Ω) and consistent within the range of resistance values measured in patients with breast cancer, with the perfect slope being 1 and y intercept 0 Ω.

### C. INTRASESSION AND INTERSESSION REPRODUCIBILITY ANALYSIS

**FIGURE 6.**
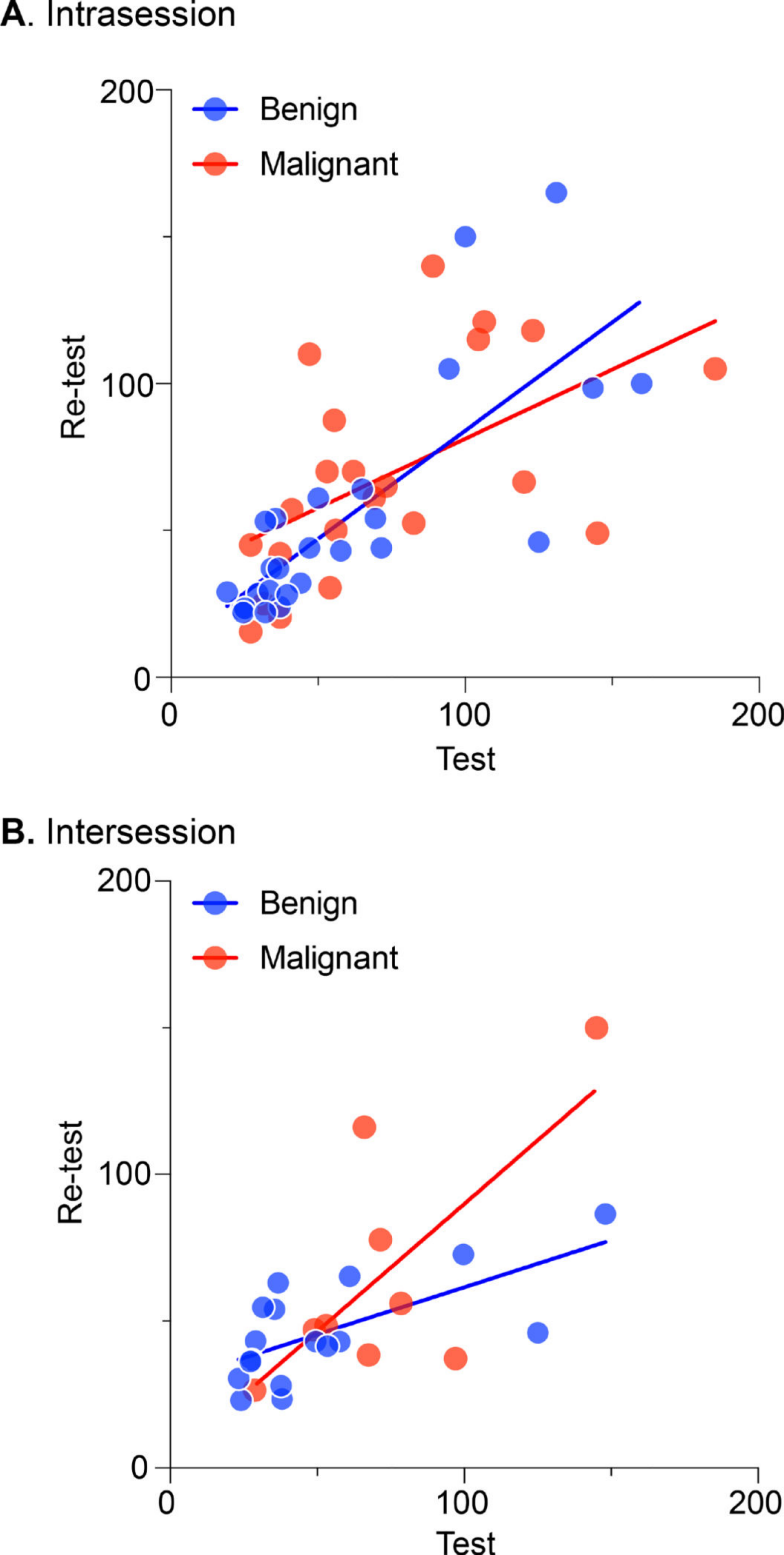
Comparison between the first and second breast lympathic measures of intrasession (A) and intersession (B) measured resistance (in kΩ). Individual values for benign (blue) and malignant (red) are plotted showing the first study on the abscissa and second study on the ordinate. ICC, intra-class correlation coefficient.

All reproducibility assessments were performed from the Sappey’s Plexus measurement points (see Figure 3). Intrasession reproducibility is shown in Figure 6A. All intersession measurements shown in Figure 6B were repeated twice at a time interval of 1 weeks after the first visit. In patients with benign and malignant cancer, the ICC intrasession values are 0.523 and 0.699, respectively, and 0.625 combined; the ICC intersession values are 0.774 and 0.554, respectively, and 0.681 combined.

### D. BENIGN VS MALIGNANT BREAST CANCER

**FIGURE 7.**
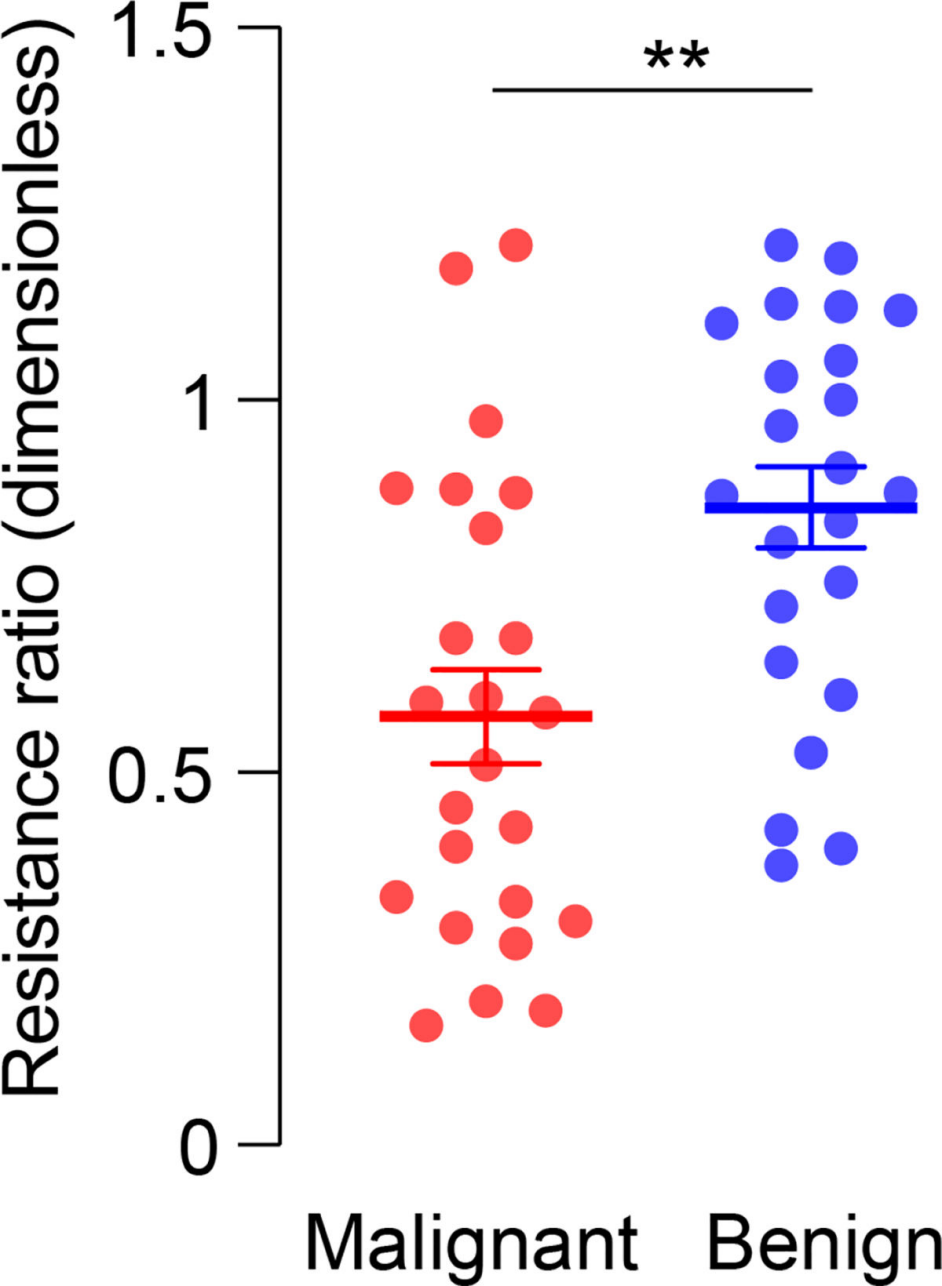
Normalized resistance values in the 2 groups of studied. T-test with Welch’s post-hoc test correction results show significant effect. **, p<0.01.

The resistance ratio between malignant and benign breast lesions are shown in Figure 7. Patients with malignant breast cancer had a lower resistance ratio compared to patients with benign breast lesions (p<0.01).

### E. TREATMENT EFFECT

**FIGURE 8.**
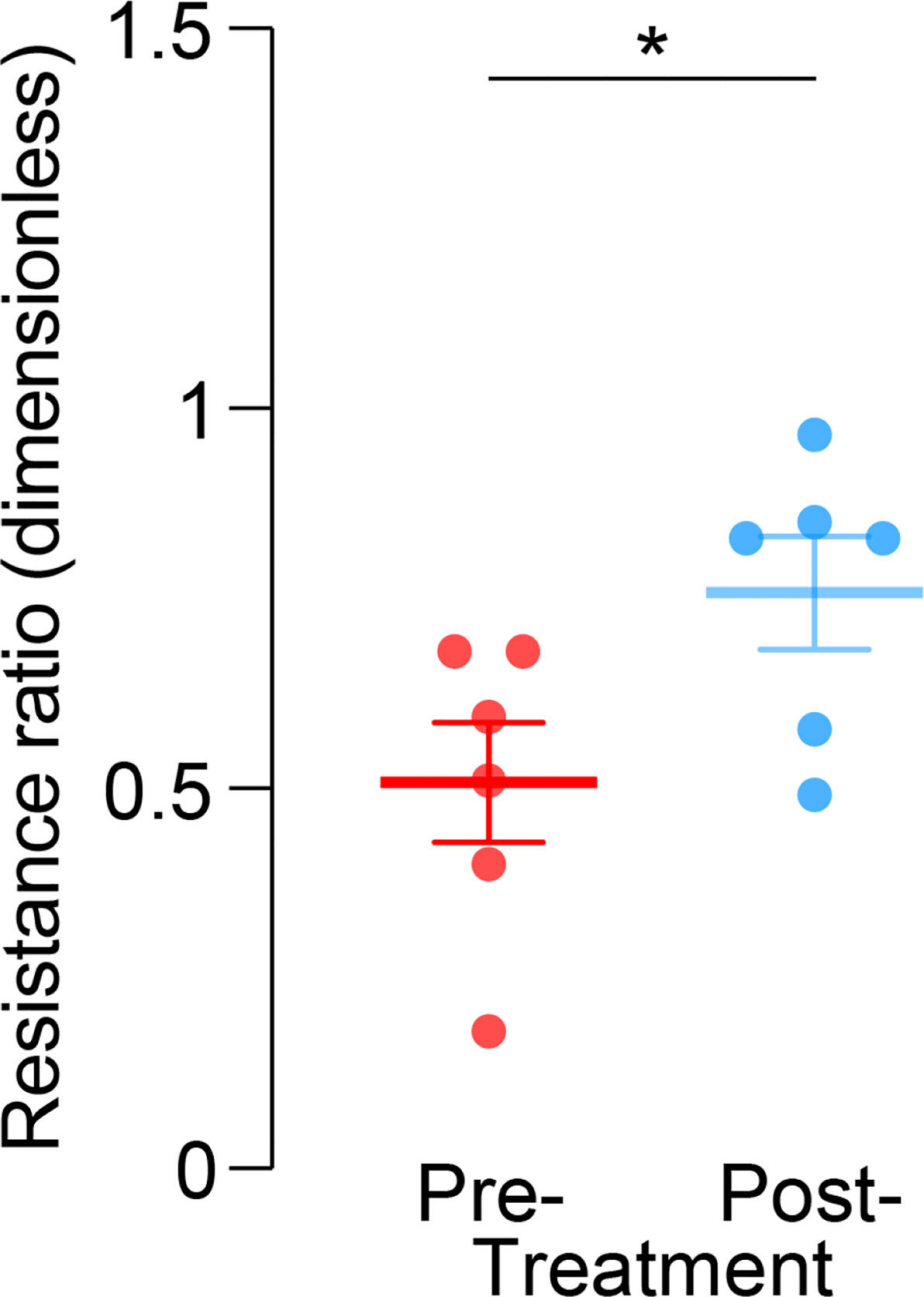
Normalized pre- and 6 months post-treatment resistance values in patients with malignant breast cancer. T-test with Welch’s post-hoc test correction results show significant effect. *, p<0.05.

Pre- and post-treatment resistance ratio are shown in Figure 8. Significant pre- and post-treatment differences were found between patients that underwent therapy (p<0.05).

### F. MACHINE LEARNING CLASSIFICATION ALGORITHM

**FIGURE 9.**
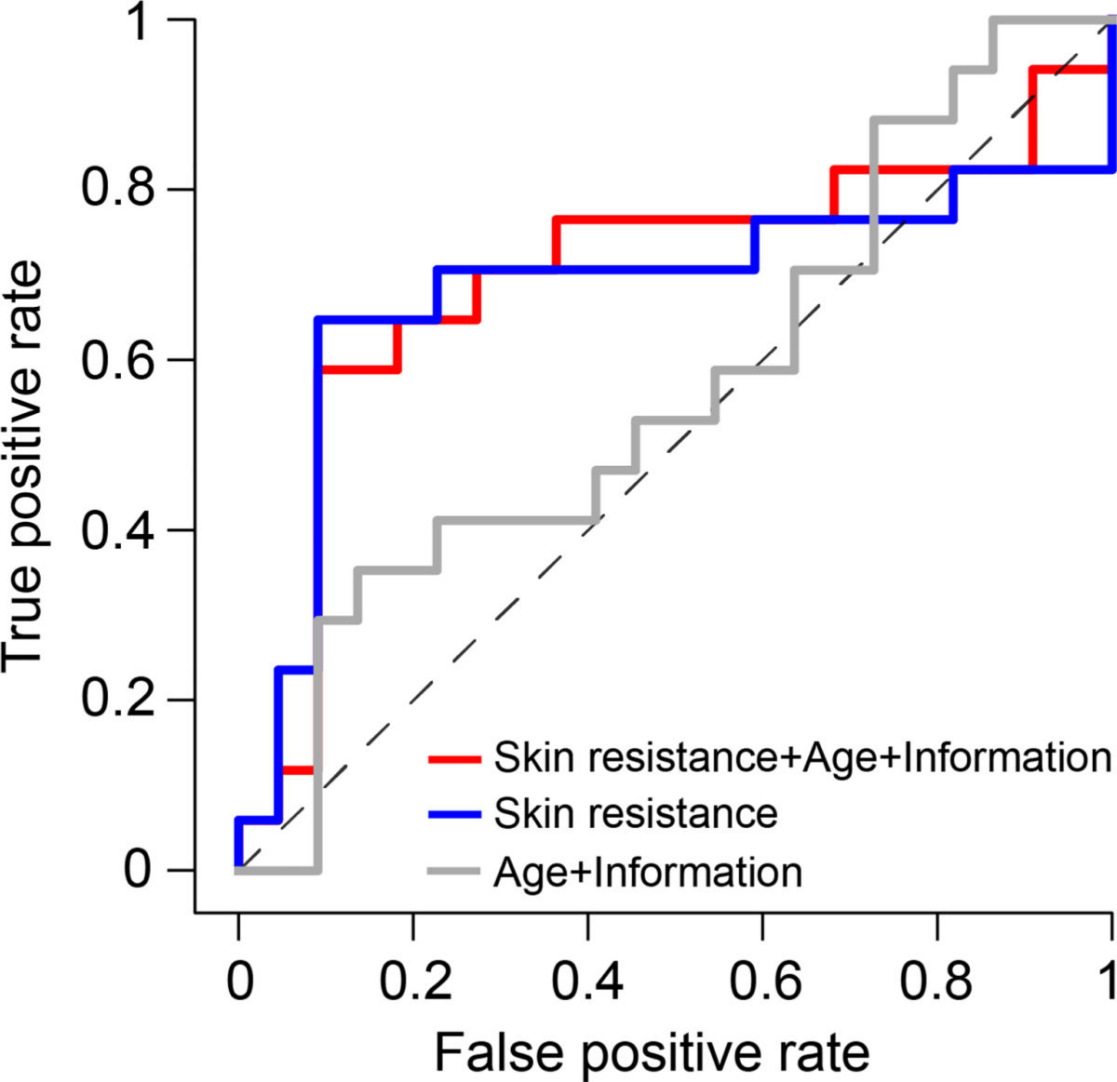
Receiver operating characteristic curves for three random forest classification models based on: (i), patients’ age and information (grey); (ii), skin resistance (blue); and (iii), skin resistance combined with patients’ age and information (red).

Receiver operating characteristic curves for the different components evaluated are shown in Figure 9. For age and patients’ information model the AUC was 0.57, sensitivity 44.59%, specificity 62.18%, PPV 47.75% and NPV 59.20% Skin resistance AUC was 0.68, sensitivity 66.35%, specificity 78.50%, PPV 70.72%, and NPV 75.13%, respectively. Finally, skin resistance combined with age and patient information was the best discriminator with AUC 0.7, sensitivity 63%, specificity 62.18%, PPV 47.75% and NPV 59.20%.

### G. TOLERABILITY

Patients’ tolerability responses are reported in Table 2. A total of N = 32 patients reported no discomfort during the measurement procedure. A minority of patient’s reported some discomfort from the measurement session (N = 15). The comments on this discomfort mentioned mild discomfort, typically around biopsied regions and/or arthritic joints. On the pain scale, majority of these patients did not rate the pain over a 5. A total of N = 45 of the patients indicated that they were willing to undergo another measurement another day versus N = 3 patients that did not agree to undergo re-measurement citing time constrains, discomfort, or no comment.

## V. DISCUSSION

In this study, skin electrical bioimpedance scanning was evaluated as a diagnostic and classification tool for breast cancer. Our work shows the intrasession reproducibility for benign and malignant cancer lesions are 0.523 and 0.699; intersession reproducibility for benign and malignant cancer lesions are 0.774 and 0.554, respectively. Our normalized results also indicate the ability to detect skin electrical changes associated with the presence of malignant versus benign breast cancer. The machine learning based classification algorithm supports the potential clinical utility, which demonstrated the capability to detect breast cancer more accurately than considering patient age and information only with an AUC 0.68, sensitivity 66.35%, specificity 78.50%, PPV 70.72% and NPV 75.13%. Finally, despite the reduced sample size (N = 6), we were able to detect pre- and post-treatment differences in patients with malignant breast cancer that received therapy. The tolerability data revealed overall good acceptance by patients and minor discomfort, who would be willing to be remeasured.

In malignant breast lesions, the normalized resistance ratio was lower than the ratio for benign patients (p<0.01, Figure 7). This indicates that the resistance at the breast lymphatic measurement points was higher than the resistance measured elsewhere in the body at the non-breast lymphatic points. This is hypothesized as a physiological change to the concentrated lymph of the Sappey’s Plexus by the malignant breast lesion increasing the measured resistance. A benign lesion would minimally change the lymphatic environment leading the resistance to be similar to the non-breast lymphatics. In the pre- and post-treatment data, we found that patients treated for a malignant breast lesion had a higher normalized resistance ratio after treatment (p<0.05, Figure 8). This suggests that before treatment was received the breast lymphatic measurement was higher in resistance than the non-breast lymphatic measurements. After treatment, however, resistance measured on breast lymphatic became lower and similar to the rest of the non-breast lymphatic regions. This may be due to the removal of the malignant tumor and treatment of the patient such that the Sappey’s Plexus is no longer being altered by a malignant tumor. Importantly, no significant differences were found between post-treatment patients (Figure 8) and benign lesion patients (Figure 7).

Cancer diagnostic bioimpedance-based instrumentation varies in complexity depending on the application and measurement approach [26]. The two-electrode, DC voltage system presented in this study was not designed to measure the tumor tissue directly, but rather to measure the relatively large dielectric changes that occur in the skin from body’s systemic immune response from cancer [27]. In other bioimpedance instances, more complex electrode systems were required to focus the measurements on a specific volume inside the body or multifrequency measurements in a particular frequency range to monitor certain physiological mechanisms [28]. Compared to other bioimpedance approaches, most studies utilized alternating current to specifically analyze electrical contrast between healthy and cancerous tissue [29]–[34]. Here, instead, a direct current was applied with a two-electrode system as in [13], where the current will generally flow in the interstitial fluids below the skin surface. By measuring the skin dielectric cancer-caused changes that occur in interstitial lymph fluids in lieu of the actual lesion, the technology may be able to detect the presence of breast cancer earlier compared to other impedance-based methods detecting the tumor when already grown to a significant size. For example, the authors in [35] used AC current to image using surface electrodes the breast and obtained a sensitivity 77% and specificity 81%. However, the positive predictive value obtained was only 28%, presumably caused by the reduced benign sample size measured. Despite being a feasibility study, no tolerability or reproducibility data were provided. Whereas the sensitivity and specificity in this study are lower, comparatively, here we did not require prior magnetic resonance imaging-based tumor identification and localization information. Also, the approach presented here might be advantageous since it is based on a a physiologically meaningful parameter such as the skin resistance [36], it requires minimal operator training and it can performed at the bedside without using expensive imaging instrumentation.

In a previous study, the authors explored the efficacy of skin resistance as a diagnostic tool comparing to the conventional diagnosis of breast cancer patients [13]. Repeated skin resistance measurements were performed on predetermined zones on the hands and feet of 45 patients with breast cancer. Baseline measurements were performed first, followed by transcutaneous electrical stimulation, and then a second last measurement was performed and then differences recorded and analyzed in comparison to the first set of values. Here, instead, we sought to explore the feasibility to diagnose cancer breast without stimulating the patients in between measurements.

This work further confirms that biompedance technology can be a useful diagnostic tool, however, we do not think the value of the technology is to replace standard mammography screenings. Instead, bioimpedance may be well suited as adjunctive test to mammography very much as ultrasonography, potentially helpful to improve agreement between radiologists when mammograms are inaccurate. For example, agreement between two radiologists interpreting screening mammography films has been reported at 86% for films depicting a cancerous mass and 84% for films depicting no cancer, and sensitivity based on the combined mammographic interpretations of 84.3% [37]. However, a more recent study showed age-specific differences, being 71% in women aged 40–49 years, 85% in those aged 50–59 years, and 86% in those aged 60–69 years [38]. The use of adjunctive ultrasonography was associated with a 0.03–0.77% overall increase in the screening detection rate [39]–[41]. Importantly, the more mammograms a patient has, the more likely to have a false positive result requiring follow up tests. The chance of having false positive result after one mammogram ranges from 7–12%. After 10 yearly mammograms the chance of a false positive rises to 50–60% [42]. Since bioimpedance technology is non-radiating, it makes it well suited to be used for frequent follow-up testing in breast cancer patients and could have use in following-up mammograms where itself can increase the risk of the disease if over-used and monitor cancer recurrence [43]. Our initial results indicate the potential value of our technological approach to detect changes due to treatment, which had not been shown before.

This work has limitations. The first limitation of this study is that we did not include healthy controls, instead, we restricted ourselves to prove the value of our approach by measuring patients with malignant and benign breast lesions. The second limitation is the use of direct current for measuring skin resistance. Although direct current has been shown previously being useful in measuring cancer caused changes in interstitial lymphatic fluid [13], the use of alternating current in concentrated lymphatic regions may provide additional skin insight and should be further studied [44], [45]. The third limitation is the system’s operator dependence. The probe electrode is hand-held and thus guided by the operator, which will affect the reproducibility of the technique. Also, the operators were not blinded during the measurement unless patients had an undiagnosed lesion to be biopsied. Other confounding factors that will likely affect the skin resistance measurements other than cancer that were not modeled here include chronic health conditions, skin hydration status, differences in skin types, skin temperature, ethnicity or menstrual cycle. Another factor to take into account is the random forest algorithm developed here, it is possible that performance could be improved with other classification algorithms not evaluated here. Regardless, we suspect sensitivity and specificity improvements will likely be achieved improving the technique and accounting for confounding variables into the machine learning algorithm which ultimately could benefit radiologists by improving accuracy of diagnosis [46].

Despite these limitations, the combination of a relatively simple noninvasive bioimpedance approach, physiologically meaningful parameter such as the skin resistance measured in lymphatic regions, and processing scheme based on a machine learning algorithm was found clinically significant and suggestive of potential as a bedside diagnostic tool for breast cancer, also to detect treatment effect. These results warrant further work to address the aforementioned limitations and also to evaluate the potential of our approach to detect cancer recurrence in breast cancer survivors.

## VI. CONCLUSION

Overall, we have successfully validated a two-electrode bioimpedance approach in the clinic measuring patients diagnosed with breast cancer. Our approach was designed and demonstrated performing measurements on patients with breast cancer to both evaluate the capabilities in detecting cancer-related dielectric changes in localized skin regions and the effect of treatment. Our approach does not alter the current standard of care and it can complement standard mammography examination by providing real-time results at the bedside face-to-face with the patient. Since it does not involve ionizing radiation, it could provide clinical value by screening patients for breast cancer earlier in their life, providing a safe adjunct technology to mammography for performing frequent assessments and also to follow patients that received therapy assessing the effectiveness of therapy.

## Figures and Tables

**TABLE 1.** Distribution of clinical stages of breast cancer and histological findings.

Diagnosis	N	Stage	N	Lesion size (cm)	N

Malignant	24	0 (DCIS)	6	<2	25
Benign	23	IA	10	[2,5]	4
		IIA	1	≥5	2
		IIA/B	1	Grouped calcifications	9
		IIB	1	Enlarged lymph nodes	2
		IIIB	1	Unknown/Not specified	5
		IIIC	1		
		IV	2		
		Not provided	1		

Abbreviation: DCIS, ductal carcinoma in situ.

**TABLE 2.** Patient tolerability response.

		Patient response (N)

Discomfort	No	32
	Yes	16
Pain scale	0	0
	1	0
	2	3
	3	7
	4	1
	5	2
	6	1
	7	0
	8	0
	9	0
	10	0
	N/A	2

Remeasurement	No	3
	Yes	45
Reason	Time constraints	1
	Discomfort	1
	No comment	1

Pain scale, 0 no pain, 10 severe pain.
